# Ethyl Acetate Extract Components of Bushen-Yizhi Formula Provides Neuroprotection against Scopolamine-induced Cognitive Impairment

**DOI:** 10.1038/s41598-017-10437-4

**Published:** 2017-08-29

**Authors:** Shi-Jie Zhang, Dan Luo, Lin Li, Rui-Rong Tan, Qing-Qing Xu, Jie Qin, Lei Zhu, Na-Chuan Luo, Ting-Ting Xu, Rong Zhang, Lei Yang, Qi Wang

**Affiliations:** 1 Institute of Clinical Pharmacology, Guangzhou University of Chinese Medicine, Guangzhou, China; 2 International Center for Translational Chinese Medicine, Sichuan Academy of Chinese Medicine Sciences, Chengdu, China; 3 Department of Radiology, the Third Affiliated Hospital, Sun Yat-sen University, Guangzhou, China

**Keywords:** Alzheimer's disease, Drug development

## Abstract

Alzheimer’s disease (AD) is a multifactorial neurodegenerative disorder and there is no effective cure for this devastating disease to date. Bushen Yizhi Formula (BSYZ-F), a Chinese herbal compound, has proved to be effective for AD. In this study, we further investigate the effective part of BSYZ-F, ethyl acetate extract components of BSYZ-F (BSYZ-E), protects scopolamine (SCOP)-induced cognitive impairment, which shows a similar effect to BSYZ-F. We also find that BSYZ-E could protect against SCOP-induced cholinergic system dysfunction. In neuron function level, BSYZ-E remarkably elevates protein levels of nerve growth factor (NGF) and brain-derived neurotrophic factor (BDNF). BSYZ-E also significantly mitigates SCOP-induced apoptosis, oxidative stress and nitrosative stress. Conclusively, BSYZ-E, the effective part of BSYZ-F, can provide neuroprotection against SCOP-induced cognitive impairment through a multifunctional strategy. These findings suggest that BSYZ-E might be developed as a therapeutic drug for AD by targeting multiple pathways of the pathogenesis.

## Introduction

Alzheimer’s disease (AD) is a chronic neurodegenerative brain disorder. AD is characterized by progressive loss of neurons mainly in hippocampus and cortex, which results in dysfunctions of cognition and emotion^1^. AD affects people aged 65 and older most commonly^2, 3^. Developed regions have become “aged society,” and the number of adults with AD is increasing^4^. The main pathological features of AD are extracellular deposits of amyloid β-proteins, neurofibrillary tangles, neuronal injury and synapse loss^3, 5^. However, the exact etiology of AD is still controversial. The cholinergic hypothesis of AD is well established^6^, which implied that the cholinergic system is important for learning and memory processes^7^. In clinical, donepezil (DON) and galantamine, which could elevate the level of acetylcholine (ACh), are now used for AD therapy^8, 9^. However, there are still some limitations, such as low efficacy, adverse effects for the long-term use^10^. Since AD is a multi-factorial disease of the central nervous system^11^, multi-component and multi-target drugs, such as traditional Chinese medicine, might be useful for AD^12, 13^.

Bushen-Yizhi formula (BSYZ-F), a traditional Chinese herbal compound composed of common Cnidium fruit, tree peony bark, ginseng root, Radix Polygoni Multiflori Preparata, barbary wolfberry fruit and Fructus Ligustri Lucidi, could increase mini-mental state examination (MMSE) scores of AD patients^14^. Our previous studies have shown that BSYZ-F could modulate cholinergic pathway, NGF signaling and anti-apoptosis in ibotenic acid (IBO)-treated rat^15^. In addition, BSYZ-F could ameliorate oxidative stress and alleviate apoptotic cell death in SCOP-treated mice^16^. These findings indicated that BSYZ-F is promised to be a potential anti-Alzheimer’s drug. However, the constituents of BSYZ-F are complex, such as paeoniflorin, 2,3,5,4-tetrahydroxylstilbene-2-O-β-D-glucoside, paeonolum, ginsenoside Rg1, ginsenoside Rb1, imperatorin, osthole, and oleanic acid, etc.^16^ In this study, we further extracted BSYZ-F by using different organic solvents to study the effective components of BSYZ-F.

Since the degeneration of cholinergic neurons is believed to be one of the leading causes of AD^17–20^, we employed a classical experimental model, scopolamine (SCOP)-treated memory disturbance model, to mimic AD-induced dementia. We found that ethyl acetate extract components of Bushen-Yizhi Formula (BSYZ-E) could protect against SCOP-induced cognitive impairment, which might be the effective part of BSYZ-F. In addition, BSYZ-E could improve cholinergic system and synaptic function. Simultaneously, BSYZ-E could also ameliorate oxidative stress and apoptosis in both hippocampus and cortex.

## Results

### HPLC analysis of BSYZ-E

**Figure 1:**
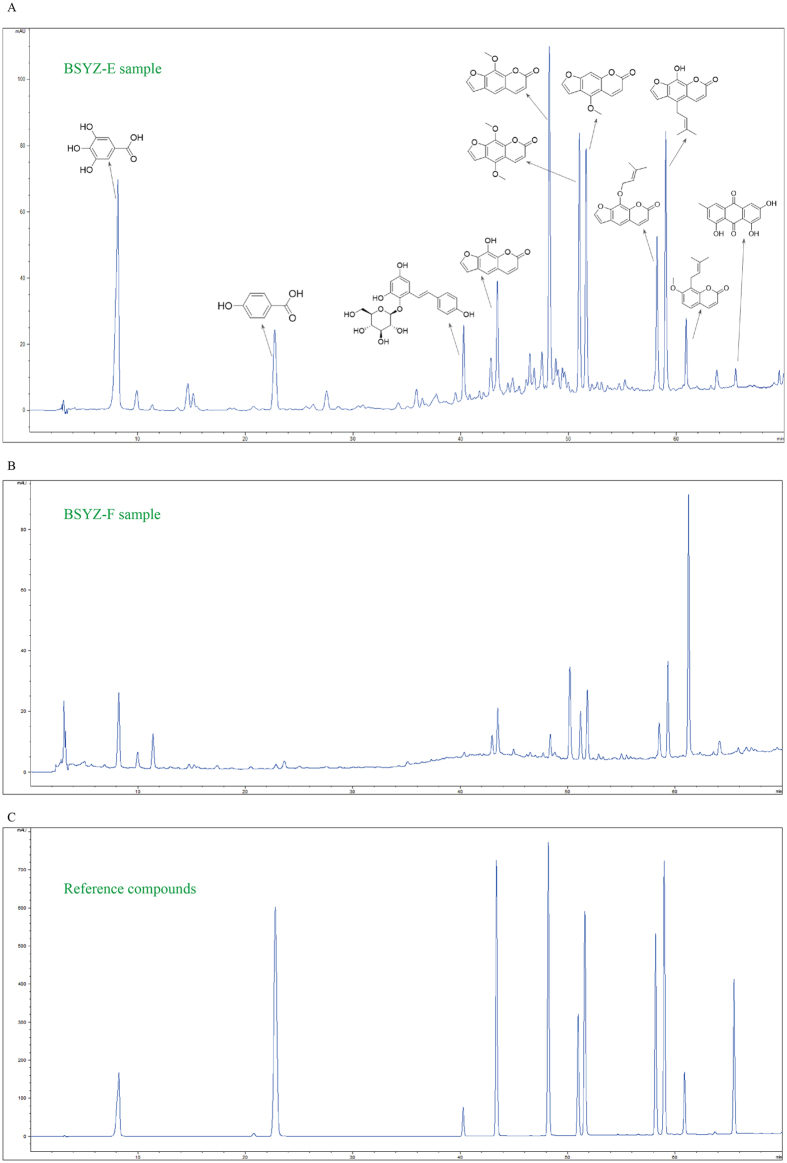
Chemical standardization of BSYZ-E analyzed by HPLC. BSYZ-E (**A**), BSYZ-F (**B**) and reference compounds (**C**) were analyzed.

Firstly, we extract BSYZ-F by using different organic solvents (petroleum ether, ethyl acetate, n-butanol and ethanol) to ensure the effective components of BSYZ-F. Morris water maze result shows that the ethyl acetate extract components of BSYZ-F (BSYZ-E)-treatment group is better than other groups (Fig. S1). The components of BSYZ-E are chemically characterized by HPLC analytical method. As shown in Fig. 1A, eleven compounds of main peaks of HPLC maps are identified as Gallic acid (Peak 1), 4-Hydroxybenzoic acid (Peak 2), 2,3,5,4’-tetrahydroxylstilbene-2-O-β-D-glucoside (Peak 3), Xanthotoxol (Peak 4), methoxsalen (Peak 5), isopimpinellin (Peak 6), bergapten (Peak 7), imperatorin (Peak 8), prangenidin (Peak 9), osthole (Peak 10) and emodin (Peak 11) respectively, by comparison with reference compounds (Fig. 1C). Due to the intermediate polarity of ethyl acetate, the compounds of middle polarity are extracted from BSYZ-F and gathered in BSYZ-E (Fig. 1B). The polar compounds in BSYZ-F, such as Paeoniflorin, Ginsenoside Rg1 etc, can not be extracted by ethyl acetate.

### BSYZ-E prevents SCOP-induced learning and memory impairments

**Figure 2:**
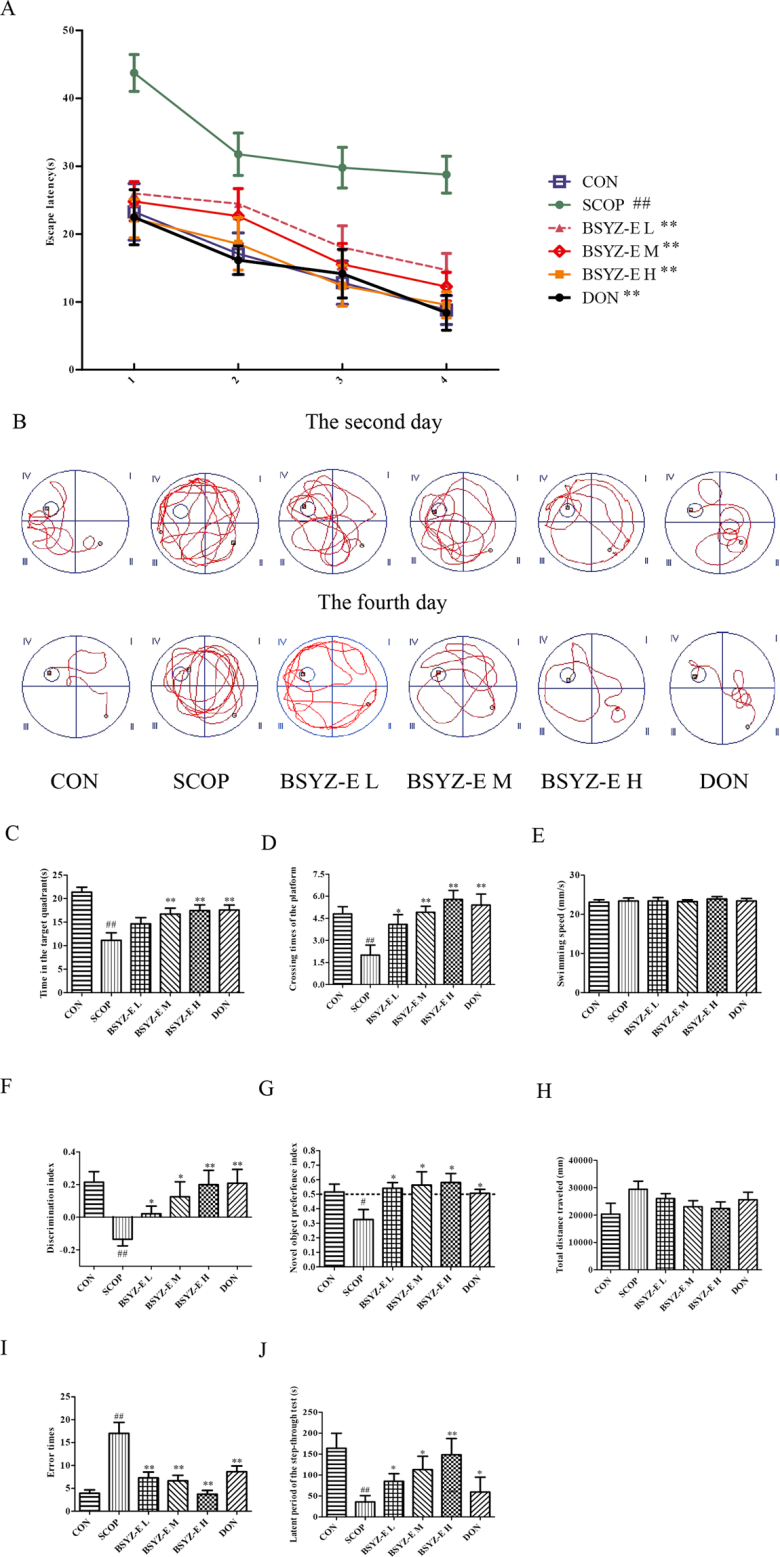
BSYZ-E prevents SCOP-induced learning and memory impairments. The Morris water maze test was performed. Escape latency of four consecutive days’ test (**A**), the swimming tracks (**B**), time spent in the target quadrant (**C**) and the crossing times of the platform (**D**) were shown. Novel object recognition test was performed. Discrimination index (**E**), novel object preference index (**F**), total distance travelled (**H**) were shown. Passive-avoidance test was performed. Error times (**I**) and the latent period of the step-through test (**J**) were shown. Experimental values were expressed as means ± SEM. ^*#*^*p* < 0.05, ^*##*^*p* < 0.01 versus CON group. *p < 0.05, **p < 0.01 versus SCOP group.

In order to investigate whether BSYZ-E could protect against SCOP-induced learning and memory impairments, we perform the Morris water maze test, novel object recognition test and passive-avoidance test. The effect of BSYZ-E on spatial memory is assessed by using the Morris water maze test. BSYZ-E L group (1.46 mg/kg), BSYZ-E M group (2.92 mg/kg), BSYZE-H group (5.84 mg/kg) and DON group (3 mg/kg donepezil) are not affect the average swimming speed compared with CON group (*p* < 0.05, Fig. 2E). As shown in Fig. 2A, the swimming time for mice to find the platform (escape latency) is reduced progressively during the four training days. The SCOP-treated mice show a significant longer escape latency than CON group (*p* < 0.01) from the first to fourth day. The result suggests that intraperitoneal injection with SCOP induce the impairment of spatial memory. After treated with BSYZ-E and DON, mice exhibit a significantly improved performance of the escape latency (*p* < 0.01). The swimming tracks of mice of each group of the second and fourth days are shown in Fig. 2B. On the second day, the mice swim aimlessly to find the hidden platform. On the fourth day, the swimming track of SCOP group is still complex. CON group swim to the platform directly. Similar performances are showed in BSYZ-E and DON groups. In spatial probe trial, there is a significant difference in time spend in the target quadrant and the crossing platform times between CON group and SCOP group (*p* < 0.01, Fig. 2C,D). However, compared to SCOP group, time spend in the target quadrant and the crossing times of four groups (BSYZ-E L, M, H groups and DON group) are increased (*p* < 0.05, *p* < 0.01, *p* < 0.01 and *p* < 0.01, respectively, Fig. 2C,D). In the novel object recognition test, SCOP-treated mice show significantly lower level of discrimination index (*p* < 0.01, Fig. 2F) and novel object preference index (*p* < 0.05, Fig. 2G) than CON group. However, BSYZ-E and DON-treated mice show significantly higher levels of these two index numbers than SCOP-treated mice (Fig. 2F,G). The total travelled distance does not differ among the groups (Fig. 2H), which indicates that the different recognition indexes of new object over the groups are not related to motor disability. In the passive-avoidance test, SCOP-treatment increases the number of trials to acquisition criterion (*p* < 0.01, Fig. 2I) and decreases the latent period of the step-through test (*p* < 0.01, Fig. 2J) significantly than CON. While BSYZ-E- and DON-treatment reverse these changes. These findings imply that treatment with BSYZ-E is beneficial for the SCOP-induced cognitive impairment.

### BSYZ-E protects against cholinergic system dysfunction in SCOP-treated mice brain

**Figure 3:**
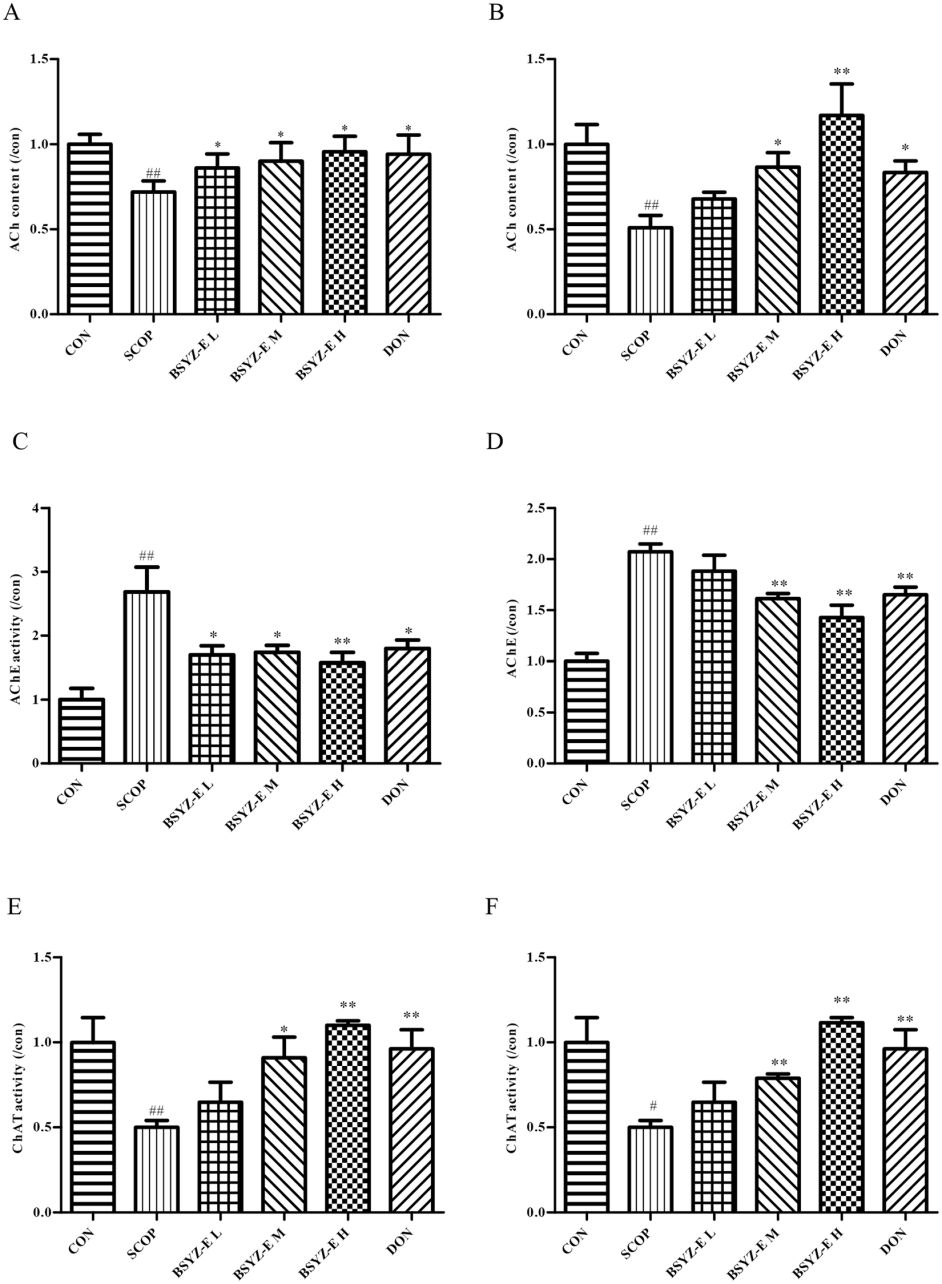
BSYZ-E protects against dysfunction of cholinergic system in SCOP-treated mice brain. ACh content (**A,B**), ChAT activity (**E,F**) and AChE activity (**C,D**) were measured in the hippocampus and cortex. Experimental values were expressed as means ± SEM. ^*#*^*p* < 0.05, ^*##*^*p* < 0.01 versus CON group. *p < 0.05, **p < 0.01 versus SCOP group.

To illuminate the potential mechanism of BSYZ-E, we detect the indexes of cholinergic system. As shown in Fig. 3A,B, SCOP causes a remarkable decrease of acetylcholine (ACh) contents in both hippocampus and cortex. However, BSYZ-E and DON significantly increase the ACh contents. The activities of acetylcholinesterase (AChE) in SCOP-treated group are increased sharply in both hippocampus and cortex (Fig. 3C,D). Whereas, BSYZ-E and DON decrease the activities of AChE significantly. The choline acetyltransferase (ChAT) activities are significantly elevated in both hippocampus and cortex of SCOP group, which are decreased remarkably in BSYZ-E and DON groups (Fig. 3E,F). These results indicate that BSYZ-E could protect against SCOP-induced cholinergic dysfunction.

### BSYZ-E improves the neuron function in SCOP-treated mice

**Figure 4:**
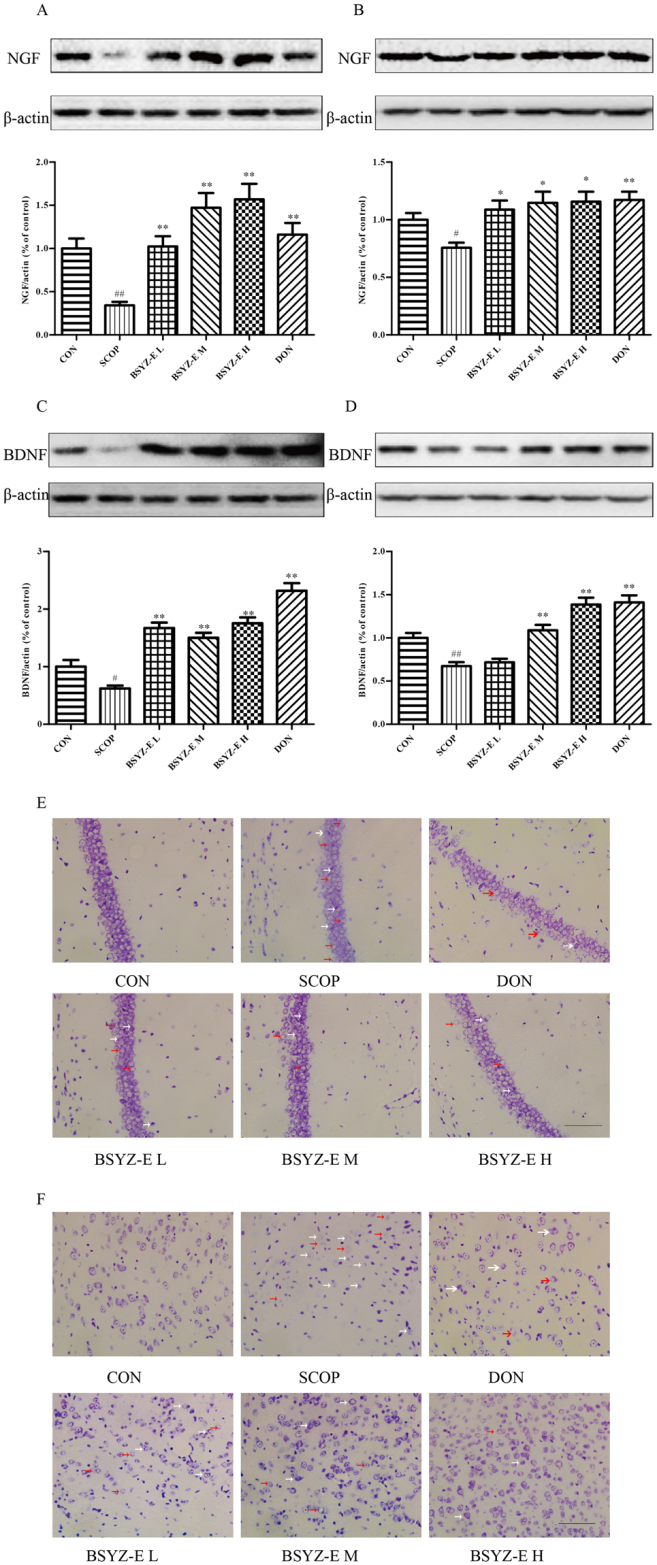
BSYZ-E improves the neuron function in SCOP-treated mice. NGF (**A,B**) and BDNF (**C,D**) protein levels were detected by Western Blotting in the hippocampus and cortex. Cell death (white arrow) and apoptotic body (red arrow) in both CA1 area (**E**) and cortex area (**F**) were shown. Scale bar: 100 *μ*m. Experimental values were expressed as means ± SEM. ^*#*^*p* < 0.05, ^*##*^*p* < 0.01 versus CON group. *p < 0.05, **p < 0.01 versus SCOP group.

Nerve Growth Factor (NGF) and brain-derived neurotrophic factor (BDNF) belongs to a group of nerve growth factors. To indentify whether BSYZ-E could up-regulate neurotrophins expressions, we detect the levels of NGF and BDNF proteins. SCOP decreases the protein levels of NGF and BDNF, while BSYZ-E and DON increase these protein levels in both hippocampus and cortex (Fig. 4A–D). Nissl staining showed that SCOP increased the number of cell death (white arrow) and apoptotic body (red arrow). In detail, SCOP reduces different thicknesses of pyramidal cell layers and density of healthy neuron cells in CA1 area of hippocampus (Fig. 4E). Meanwhile, SCOP also results in typical neuropathological changes, including nissl bodies loss and nucleus shrinkage or disappearance in cortex (Fig. 4F). After administration of BSYZ-E or DON, these SCOP-induced neuron damages are attenuated. These results indicate that BSYZ-E could protect against SCOP-induced neuron dysfunction.

### BSYZ-E alleviates apoptosis in SCOP-treated mice brain

**Figure 5:**
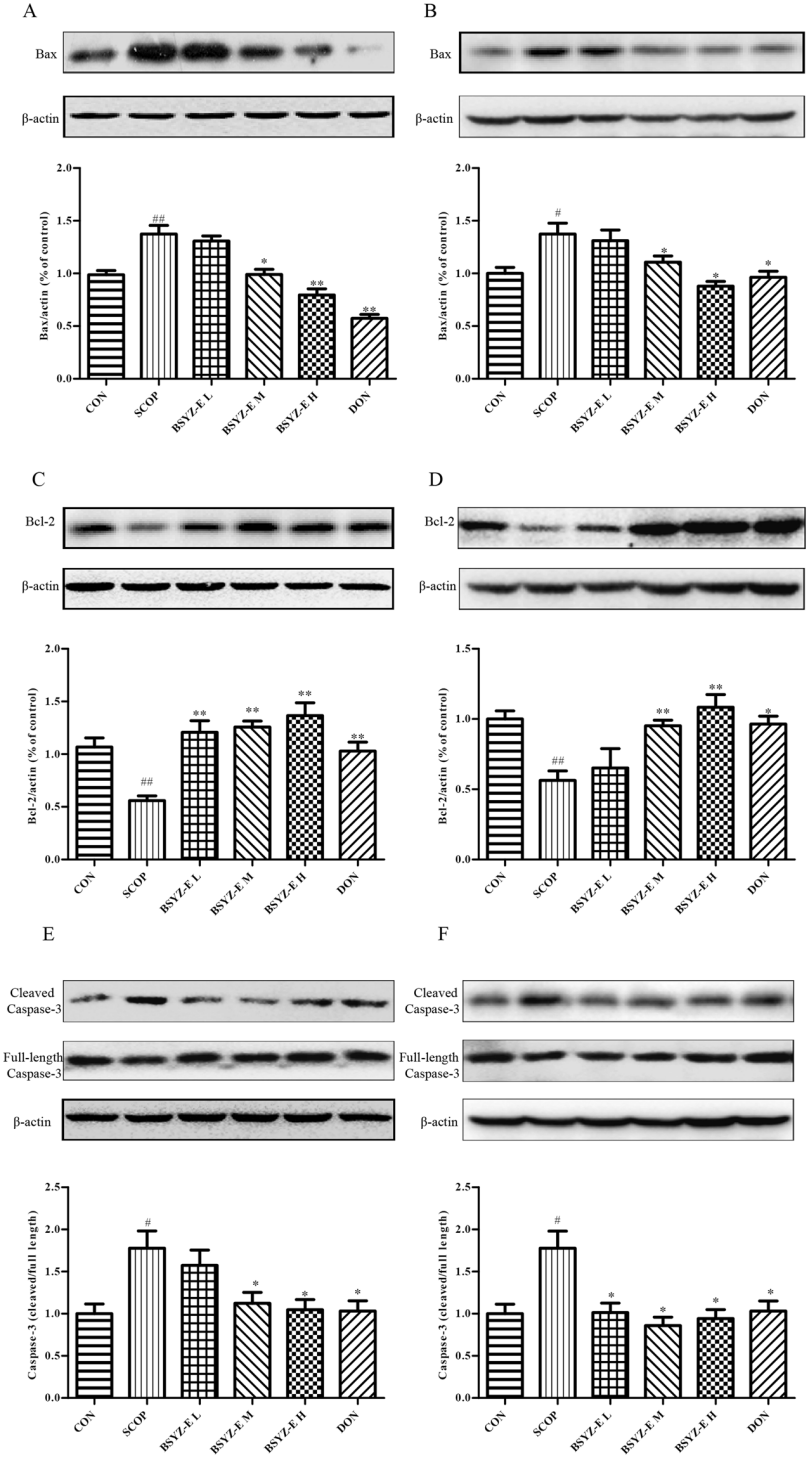
BSYZ-E alleviates apoptosis in SCOP-treated mice brain. Bax (**A,B**), Bcl-2 (**C,D**) and cleaved Caspase-3 (**E,F**) protein levels are detected by Western Blotting in the hippocampus and cortex. Experimental values were expressed as means ± SEM. ^*#*^*p* < 0.05, ^*##*^*p* < 0.01 versus CON group. *p < 0.05, **p < 0.01 versus SCOP group.

**Figure 6:**
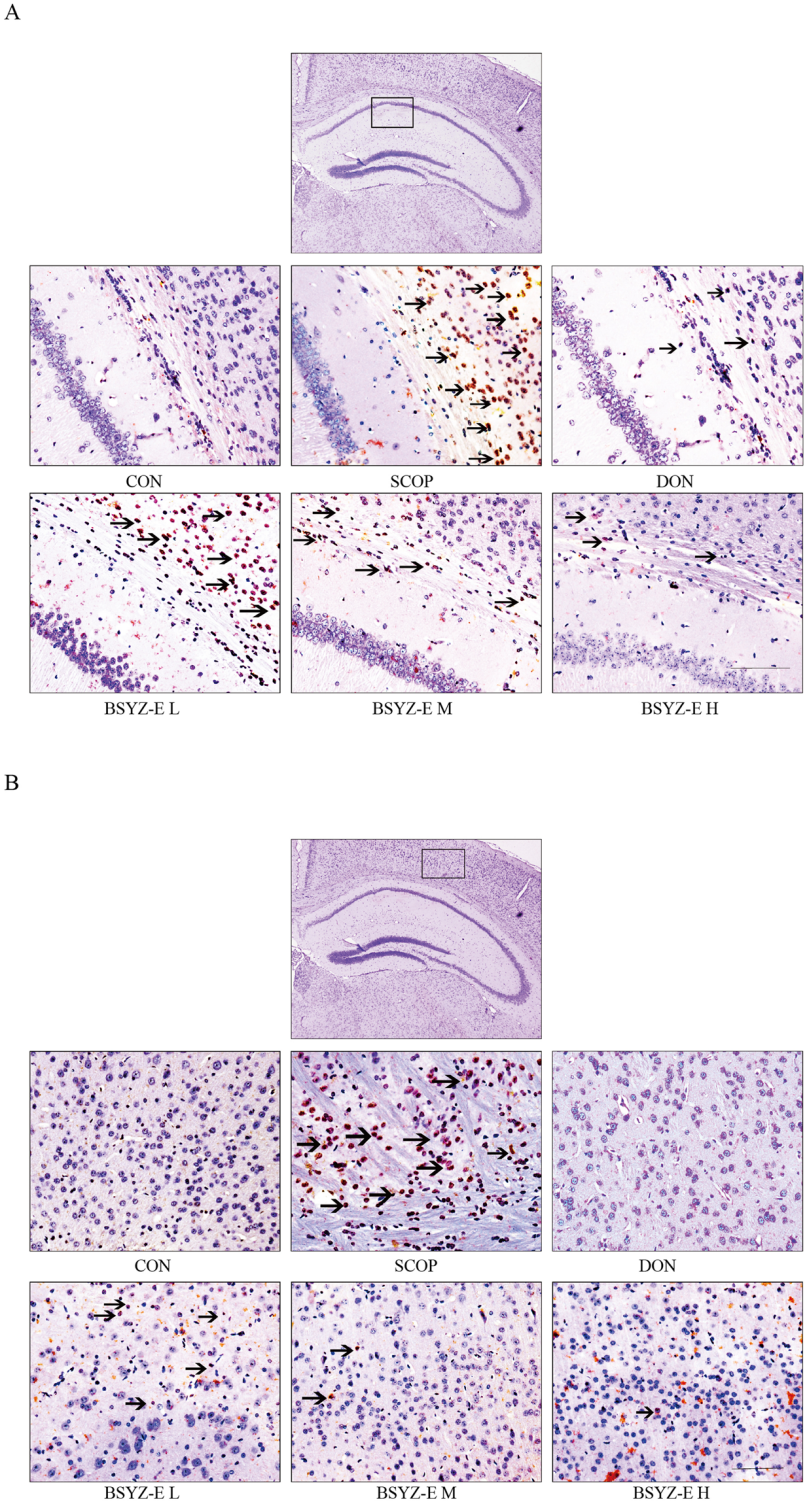
TUNEL staining. Neuronal apoptosis in SCOP-treated mice CA1 area (**A**) and cortex area (**B**) were shown. Red arrows showed the neuronal apoptosis cell death. Scale bar: 100 *μ*m.

We further investigate the apoptosis in SCOP-treated mice. As shown in Fig. 5A–F, SCOP remarkably increases the proapoptotic proteins Bax and cleaved Caspase-3 expression and decreased the expression of Bcl-2 in both hippocampus and cortex. BSYZ-E and DON significantly upregulate the Bcl-2 protein expression and downregulates the Bax and cleaved Caspase-3 protein expressions. TUNEL staining shows that the neuronal apoptosis is prominently increased in both hippocampus and cortex of SCOP-treated mice. BSYZ-E and DON markedly attenuate the neuronal apoptosis induced by SCOP (Fig. 6A,B). These results indicate that BSYZ-E could alleviate SCOP-induced neuronal apoptosis.

### BSYZ-E relieves oxidative stress and inducible nitric oxide synthase (iNOS) in SCOP-treated mice brain

**Figure 7:**
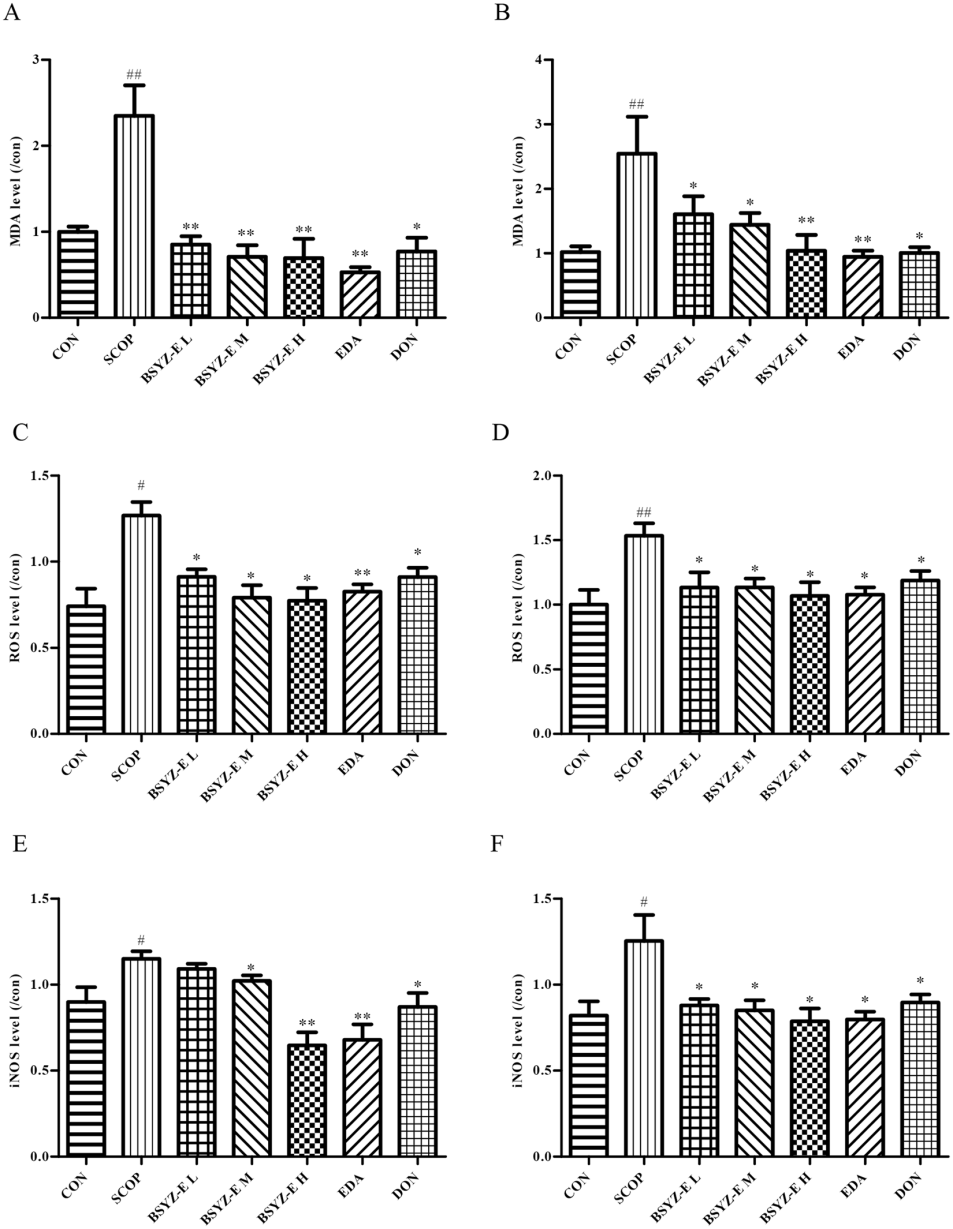
BSYZ-E relieves oxidative stress and inducible nitric oxide synthase (iNOS) in SCOP-treated mice brain. The levels of MDA (**A,B**), ROS (**C,D**) and iNOS(**E**,**F**) were detected in both hippocampus and cortex. Experimental values were expressed as means ± SEM. ^*#*^*p* < 0.05, ^*##*^*p* < 0.01 versus CON group. *p < 0.05, **p < 0.01 versus SCOP group.

**Figure 8:**
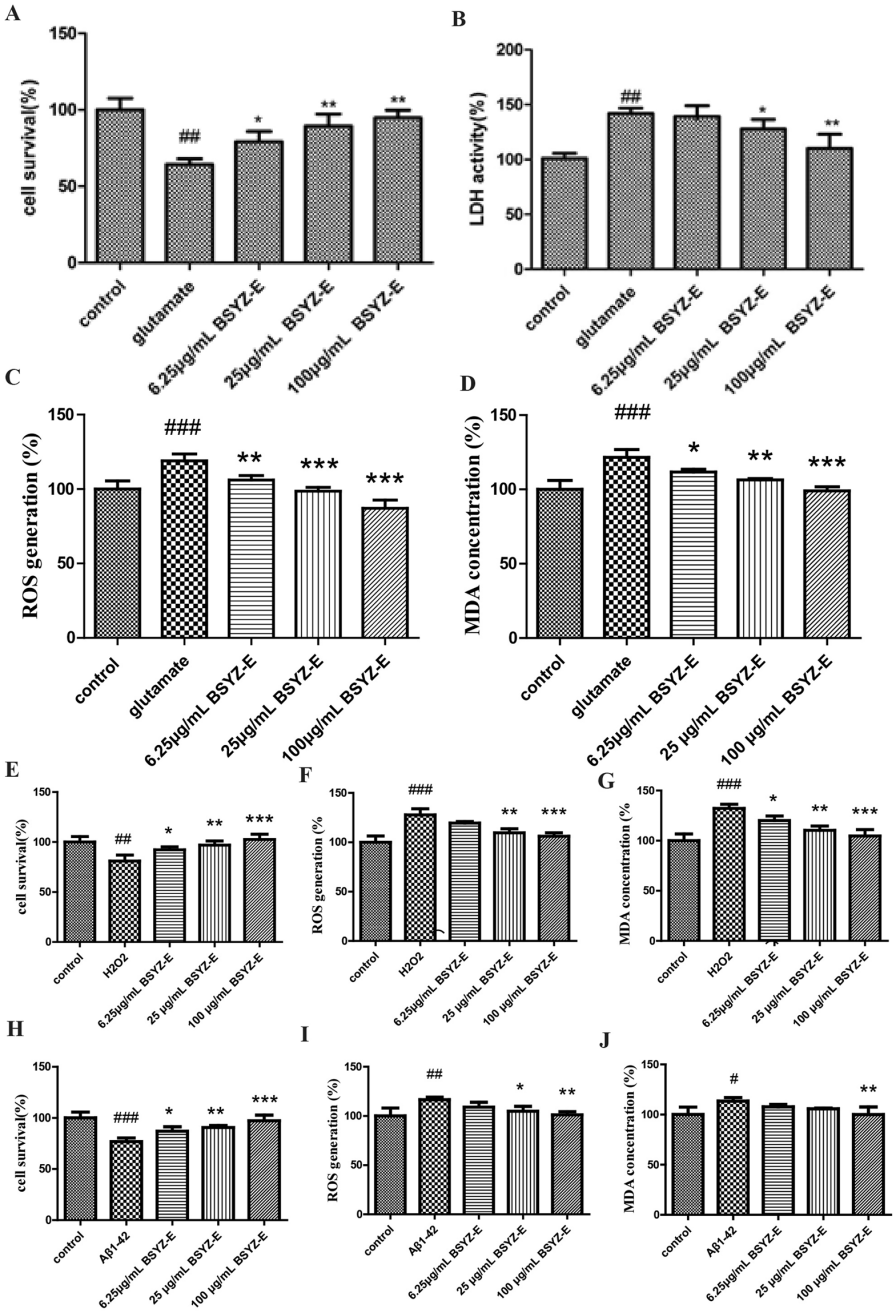
BSYZ-E protects glutamate, H_2_O_2_ or Aβ1-42-induced cell damage in PC12 cells. Cell survival rate, LDH activity, ROS and MDA levels were measured in PC12 cells. Experimental values were expressed as means ± SEM. ^#^*p* < 0.05, ^##^*p* < 0.01, ^###^*p* < 0.001 versus control group. *p < 0.05, **p < 0.01, ***p < 0.001 versus glutamate, H_2_O_2_ or Aβ1-42 group.

We also test the oxidative stress status in both hippocampus and cortex (Fig. 7A–D). The levels of MDA and ROS are significantly higher in the SCOP group when compared with CON group, whereas BSYZ-E, DON and edaravone (EDA) treatment reverse the changes. EDA also improves the SCOP-induced learning and memory impairments (Fig. S2). The level of iNOS is also increased in both hippocampus and cortex of SCOP group (Fig. 7E,F). BSYZ-E and DON decrease the levels of iNOS in SCOP-treated mice. We also perform some cell studies to prove the neuroprotective effect of BSYZ-E (Fig. 8). We employ three cell models, glutamate, H_2_O_2_ or Aβ1-42-treated PC12 cell model, to study the neuroprotective effect of BSYZ-E. 6.25 μg/mL, 25 μg/mL or 100 μg/mL BSYZ-E is added to the culture medium of PC12 cells for 24 h. After BSYZ-E pretreatment, glutamate, H_2_O_2_ or Aβ1-42 is added to culture medium of PC12 cells respectively. The survival of PC12 cells is detected by MTT. The oxidative stress in PC12 cells is also been detected. Results show that glutamate, H_2_O_2_ or Aβ1-42 decreases the survival rate of PC12 cells and increases LDH activity, ROS and MDA levels. While, BSYZ-E improves the survival rate of glutamate, H_2_O_2_ or Aβ1-42-treated PC12 cells in a dose-dependent manner. And LDH activity in cell culture supernatant decreases with the intervention of BSYZ-E. The levels of ROS and MDA are also decreased. These results indicate that BSYZ-E could protect against SCOP-induced oxidative stress and nitrosative stress.

## Discussion

In this study, we evaluated the neuroprotective effect of BSYZ-E, the effective part of BSYZ-F, in SCOP-treated mice. Treatment with BSYZ-E significantly improves SCOP-induced cognitive impairment. In mechanism study, we find that BSYZ-E prominently improves the cholinergic function and prevents SCOP-induced synapse injury. Meanwhile, the anti-apoptosis and antioxidative effects of BSYZ-E are also verified. These experimental evidences show that BSYZ-E might be a multifunctional drug for AD.

BSYZ-F Formula contains a variety of complex components. Hence, we study different types of BSYZ-F extracts to investigate the effective components of BSYZ-F. We verify that the ethyl acetate extract components (BSYZ-E) is the most effective extract of BSYZ-F. Gallic acid, 4-Hydroxybenzoic acid, 2,3,5,4′-tetrahydroxylstilbene-2-O-β-D-glucoside, Xanthotoxol, methoxsalen, isopimpinellin, bergapten, imperatorin, prangenidin, osthole and emodin are identified as the most abundant monomer compounds in BSYZ-E. These small molecules have been shown to have anti-cholinesterase, anti-apoptotic, anti-oxidative effects and be related with synaptic growth^21–27^. All of the compounds from BSYZ-E are the pharmacodynamic substance foundation.

Cholinergic malfunction is one of leading cause of deterioration of cognitive processing in AD, which is found in the aged and demented central nervous system^28–31^. In addition, blocking cholinergic mechanism in young subjects can artificially induce loss of memory exist^32^. Scopolamine (SCOP), one of cholinergic blocking-agents, can result in a “scopolamine dementia”^18, 20^. In this study, we employ a mimic AD model, SCOP-treated mice model, to evaluate the anti-AD activity of BSYZ-E. Behavioural tests show that BSYZ-E could protect against SCOP-induced cognitive impairment. SCOP primarily blocks Acetylcholine (ACh) receptors, causes cognitive impairment and then results in anticholinergic activities^33^. Abundant studies found the reduction of ACh levels in human brains afflicted with AD^34, 35^. Acetylcholinesterase (AChE) is a kind of hydrolytic enzyme that rapidly hydrolyzes Ach^36^. The choline acetyltransferase (ChAT) is an important symbol of cholinergic system, and is a synthetic enzyme that associated with the synthesis of Ach^37^. In current study, we find that SCOP decreases ACh content and ChAT activity, increases activity of AChE in both hippocampus and cortex, which indicate that the dysfunction of cholinergic nervous system might facilitate the process of cognitive impairment. While BSYZ-E reverses these changes significantly. Positive contrast drug, donepezil (DON), shows a similar effect. Thus, BSYZ-E could provide neuroprotective effect against SCOP-induced cholinergic system dysfunction.

It is reported that SCOP could induce neuron injury in mice brain^38^. Nerve growth factor (NGF) and brain-derived neurotrophic factor (BDNF) play an important part in neurogenesis and synaptogenesis^26, 39–41^. We find that BSYZ-E and DON could increase the protein levels of NGF and BDNF and protect synaptic structure. The neuroprotective effect of BSYZ-E might be mediated by enhancing synaptic function and stimulating the levels of nerve growth factors.

Studies have shown that SCOP could induce oxidative stress and promote apoptosis^42–44^. And oxidative stress and nitrosative stress contribute to AD pathogenesis^3, 34, 44^. Results show that the levels of MDA, ROS and iNOS are significantly increased in the SCOP group, whereas BSYZ-E and DON treatment reverse the changes. Edaravone, an oxygen radical scavenger, shows the similar effect. In addition, glutamate, H_2_O_2_ or Aβ1-42-treated PC12 cell model also proves the anti-oxidative stress effect of BSYZ-E. Apoptosis, a programmed cell death, leads to brain shrinkage in AD brains^12, 25^. Our results show that BSYZ-E remarkably downregulates the apoptotic index Bax/Bcl2 and cleaved Caspase-3 expressions in hippocampus and cortex of SCOP-treated mice. In addition, TUNEL staining shows that BSYZ-E markedly attenuates the SCOP-induced neuronal apoptosis. These results suggest that the protective effect of BSYZ-E against SCOP-induced cognitive injury is related with inhibiting oxidative stress and apoptosis.

In conclusion, we tentatively put forward that BSYZ-E provides neuroprotection against SCOP-treated cognitive dysfunction, and the mechanisms is multiple. As one kind of multi-target strategies, BSYZ-E might be a potential anti-AD drug. However, the further neuroprotective mechanisms and clinical trial of BSYZ-E are still need to be investigated.

## Materials and Methods

### Preparation of BSYZ-E

The extracts of BSYZ-F (common Cnidium fruit, tree peony bark, ginseng root, Radix Polygoni Multiflori Preparata, barbary wolfberry fruit and Fructus Ligustri Lucidi at a ratio of 3:3:2:2:2:2) were purchased from the Guangzhou medicine company and authenticated by Doctor Shixiu Feng, pharmacognosist of Shenzhen Fairy Lake Botanical Garden. All of these accorded with the standard described in the Pharmacopoeia of People’s Republic of China. BSYZ prescription compounds were cut into small pieces, boiled in 8 volumes of distilled water at 80 °C three times (1.5 h) and then concentrated. The mixture was steeped with 2 volumes of absolute ethyl alcohol for overnight stratification. After filtrating, the residue was extracted with ethanol (67% v/v) twice. The filtrate was concentrated under reduced pressure by rotary evaporator to afford crude water extract. BSYZ-E was extracted from the crude water extract by using ethyl acetate 8 times and distilled ethyl acetate after reaction. The crude extract was dried under vacuum to yield a brown, sticky fraction. Finally, 1 g of BSYZ-E was determined to contain 113.76 g of crude herbs. The fraction was stored in at 4 °C before being resolved with distilled water for usage, according to the standard of 1 g/ml (w/v).

### HPLC analysis

BSYZ-E was qualitatively analyzed by HPLC. Agilent 1200 liquid chromatography system (Santa Clara, USA), equipped with ELSD, a quaternary solvent delivery system, a column temperature controller, and an autosampler, was used for chromatographic analysis. BSYZ-E, BSYZ-F and the mixed standard solution were separated on Phenomenex Luna-C18 column (250 mm × 4.6 mm, 5 μm) at 40 °C. The mobile phase was composed of 0.1% formic acid in water (A) and 0.1% formic acid in methanol (B). Programmed gradient elution was performed as follow: 0–30 min, 10–30% B; 30–40 min, 30–50% B; 40–70 min, 50–100% B. The flow rate was 1 mL/min. Monitoring was performed at 254 nm with PDA detector. Chromatographic data were recorded and processed with Allchrom Plus Client/Server software.

### Animals

Male Kunming (KM) mice weighing 35–40 g obtained from the Experimental Animal Center of Guangzhou University of Chinese Medicine (Guangzhou, China). The animals were housed in a pathogen-free room under standard conditions (temperature: 22 ± 2 °C, humidity: 50–60%, 12-h light/12 - h dark cycle, light on at 8:30 am.) with free access to food and water for the duration of the study. All of the animal experiments were approved by the animal ethics Committee of Guangzhou University of Chinese Medicine, in accordance with the guide for the animal experiments, clinical studies and biodiversity rights.

### Drug administration

Scoplamine hydrobromide injection was purchased from Guangzhou Pharmaceuticals Corporation (Guangzhou, China). Donepezil (Eisai China Inc) and edaravone (Boda Pharmaceutical) were dissolved in 0.9% physiological saline. The gavage doses of BSYZ-F were 5, 10 and 20 times the clinical dosage (17.5 g dosage, 60 kg human), respectively. Since 0.88 g of BSYZ-E was determined to contain 100 g of BSYZ-F. The oral dose of BSYZ-E is 1.46 mg/kg, 2.92 mg/kg and 5.84 mg/kg. Mice were randomly sorted into the following groups, with 12 mice in each group: the vehicle control group (CON, 0.9% Saline), scoplamine group (SCOP 3 mg/kg), low dose BSYZ-E group (BSYZ-E L, SCOP 3 mg/kg + BSYZ-E 1.46 mg/kg), medium dose BSYZ-E group (BSYZ-E M, SCOP 3 mg/kg + BSYZ-E 2.92 mg/kg), high dose BSYZ-E group (BSYZ-E H, SCOP 3 mg/kg + BSYZ-E 5.84 mg/kg), Donepezil group (DON, SCOP 3 mg/kg + DON 3 mg/kg) and Edaravone group (EDA, SCOP 3 mg/kg + Edaravone 33.2 mg/kg). Animals were treated by oral gavage with Saline, BSYE-E, Donepezil or Edaravone continuously once per day. Mice underwent the behavioural tests from the 8th to the 17th day. Except CON group received saline intra-peritoneally (ip) 30 min after treatment with Saline, all mice were injected with SCOP 30 min after oral administration of BSYE-E, DON or EDA. They were taken the behavioural tests 30 min after SCOP injection.

### Morris water maze test

The Morris water maze test was conducted in the method of Morris as described previously^45^. In brief, the water maze apparatus (Guangzhou Feidi Biology Technology Co., Ltd., Guangzhou, China) was a black circular tank (diameter: 120 cm; height: 40 cm), including a black Plexiglass escape platform (diameter: 8 cm), four equal quadrants and four equivalently spaced starting stations. The pool was filled with water (temperature: 22–26 °C) dyed black to a depth of 30 cm, and the platform was submerged in the center of one of the quadrants to a depth of 1.5 cm from the water surface. On the four consecutive training days, mice was placed at one of the starting points facing the wall, and released into the pool. The swimming time to find the hidden platform from the starting point was recorded and analyzed using the record system. If the mouse failed to escape within 60 s, the swimming time was assigned as 60 s. The mice was manually guided to the platform by the experimenter, left for 20 s for recognize the location. The procedure was repeated with each mice starting in each of the four quadrants stochastically changed on each training day. On the fifth day, the hidden platform was removed, the animal was allowed to swim freely for 60 s. Swimming speed, time spent in the target quadrant and the crossing times of the platform were measured to evaluate retention of spatial memory.

### Novel object recognition test

The novel object recognition test was performed as described in a previous study with a minor modification^46^. On day 1, the animals were habituated during 5 minutes a brightly testing arena (length: 50 cm; width: 25 cm; height: 50 cm), dimensions 380 × 380 × 15 mm (length × width × height), covered with shavings. On day 2 (after 24 h), the animals were re-exposed to the box, in which 2 identical objects were affixed diagonally. Mice were allowed to explore for 5 min inside the arena. On day 3, one of the familiar objects was replaced by a novel object, and the mice were placed back to the arena for 5 min. The time spent on exploring the familiar object (Tf) and novel object (Tn) were recorded, and the records were analyzed through the computerized The novel object preference index and discrimination index obtained by the formulae were used to measure recognition memory: discrimination index = (Tn − Tf)/(Tn + Tf)^47^, novel object preference index = Tn/(Tn + Tf)^48^. The total travelled distance was also measured as a control of the test^49^.

### Passive-avoidance test

The passive-avoidance test was performed as described in a previous study with a minor modification^50^. In brief, after training, mice were placed in the lighted chamber, and 10 s later, the guillotine door was opened. The number of trials to acquisition of passive-avoidance test and the latent period of the step-through test in the dark compartment were recorded for up to 600 s.

### Brain sections and tissue preparation

Mice were sacrificed for sample collection after finishing the behavioural tests. 10 mice in each group were randomly selected, anesthetized with 10% chloral hydrate and decapitated. Brains were rapidly removed and cleaned with 0.1 M phosphate buffer (PBS, pH 7.4) on ice, hippocampus and cortex were carefully dissected from brains and stored at −80 °C until usage of other analysis. The mice were anesthetized and decapitated. Brains were submerged in 4% paraformaldehyde in 0.1 M phosphate buffer (PBS, pH 7.4) for pathomorphology and immunohistichemistry.

### Measurement of ACh level, AChE and ChAT activities

All mice were anesthetized and decapitated after the Morris water maze test immediately; hippocampus and cortex were carefully dissected frombrains for examination. All the processes were performed on ice-cold plate. Tissues were rapidly stored at −80 °C. The hippocampus and cortex tissues were homogenized with ice-cold saline. The homogenate was centrifuged at 12,000 × g for 10 min at 4 °C. The supernatant was used to detect the activity of ACH, ChAT and AChE according to the manufacturer’s instructions by using Universal Microplate Spectrophotometer (Bio-Rad, Hercules, CA, USA).

### Cell Culture and Drug Treatment

Differentiated PC12 cells, obtained from the Chinese Academia Sinica (Shanghai, China), were cultured in DMEM medium supplemented with 10% (v/v) fetal bovine serum 37 °C in a humidified atmosphere of 5% CO_2_ incubator. The differentiated PC12 cells were treated with different concentrations of BSYZ-E (6.25 μg/mL, 25 μg/mL or 100 μg/mL) for 3 h prior to exposure to glutamate, H_2_O_2_ or Aβ1–42. After 24 h, the subsequent experiments were conducted.

### MTT Assay

PC12 cells were seeded in a 96-well plate, and the next day, cells were treated with glutamate, H_2_O_2_ or Aβ1-42 for 24 h. Some cells were pretreated with BSYZ-E (6.25 μg/mL, 25 μg/mL or 100 μg/mL) for 3 h, which was followed by treating with glutamate, H_2_O_2_ or Aβ1-42 for 24 h. Subsequently, 10 μL of MTT solution was added at a final concentration of 5 mg/ml, and incubated for another 4 h at 37 °C, then the medium was removed, and 150 μL DMSO was added into each well. After that, the absorbance was measured at 570 nm with Universal Microplate Spectrophotometer (Bio-Rad, Hercules, CA, USA).

### Measurement of ROS Production

The hippocampus and cortex tissues were homogenized with ice-cold saline and centrifuged at 12,000 × g for 10 min at 4 °C. PC12 cells were collected and washed twice with ice-cold PBS before lysis. The supernatant was used to detect the levels of ROS. ROS were measured using the redoxsensitive fluorescent dye, DCFH-DA. Conversion of nonfluorescent DCFH-DA to fluorescent dichlorofluorescein (DCF) in the presence of ROS was measured on a microplate reader. Fluorescence emission intensity of DCF (538 nm) was measured in response to 485 nm excitation. The level of intracellular ROS was expressed as a percentage of control cultures incubated in DCFH-DA.

### LDH, MDA, SOD and iNOS assays

The hippocampus and cortex tissues were homogenized was centrifuged at 3,000 × g for 10 min at 4 °C and the supernatant was used to assay. PC12 cells were collected and washed twice with ice-cold PBS before lysis. LDH, SOD activity, MDA content and iNOS content were detected by using the commercial kits according to the manufacturer’s instructions by using Universal Microplate Spectrophotometer (Bio-Rad, Hercules, CA, USA).

### Nissl staining

The slides were dipped in Nissl Staining Solution (Beyotime Institute of Biotechnology, China) for 30 s, washed again with distilled water, and dehydrated through an alcohol series (dipped in 70%, 80%, 90%, and twice in 100% alcohol, for 30 s each). The sections were permeabilized with xylene and mounted with neutral resin. The background was colorless, and Nissl bodies were stained blue-purple. Images were analyzed by using a light microscope and LEICA QWin Plus (Leica Microsystems, Wetzlar, Germany).

### TUNEL Staining

The sections were washed in PBS and incubated with TUNEL reaction mixture in the dark. Further incubation with converter-POD was performed. The sections were then rinsed with PBS and stained with DAB substrate. TUNEL staining was performed using the *In Situ* Cell Death Detection kit (Roche Diagnostics GmbH, Mannheim, Germany). Images were analyzed by using a light microscope and LEICA QWin Plus (Leica Microsystems, Wetzlar, Germany).

### Western blot analysis

The hippocampus and cortex tissues were homogenized and lysed in ice-cold RIPA buffer (containing 1: 100 PMSF, 1: 100 inhibitor proteases and phosphatasescocktail) for 15 min. The lysate was centrifuged at 12,000 × g for 10 min at 4 °C. The same amount of protein (30 μL) was separated by SDS-PAGE analysis gel. Then the separated protein migrated to PVDF membranes and was blocked in 5% skim milk that dissolved in Tris-buffered saline-Tween-20 (TBST) for 1 h at room temperature. The membranes containing the protein were incubated with rabbit anti-Bax (1: 1,000, Santa Cruz, Barbara, CA, USA), rabbit anti-Bcl2 (1: 1,000, Cell Signaling Technology, Boston, MA, USA), rabbit anti-Caspase-3 (1: 1,000, Cell Signaling Technology, Boston, MA, USA), rabbit anti-Synaptophysin (1:1,000, Cell Signaling Technology, Boston, MA, USA), rabbit anti-PSD93 (1:1,000, Cell Signaling Technology, Boston, MA, USA), rabbit anti-PSD95 (1:1,000, Cell Signaling Technology, Boston, MA, USA), rabbit anti-BDNF antibody (1:1000, Abcam), rabbit anti-NGF antibody (1:1000, Abcam) and mouse anti-β-actin (1: 1,000, Sigma-Aldrich, St. Louis., MO, USA) overnight at 4 °C. Then the membrane was incubated with horseradish peroxidase conjugated anti-rabbit (Cell Signaling Technology, Boston, MA, USA) or anti-mouse (Cell Signaling Technology, Boston, MA, USA) IgG antibody (1: 1,000) for 1 h at room temperature. The membrane was visualized by using a superenhanced chemiluminescence reagent (ECL; Applygen Technologies Inc., Beijing, China).

### Statistical analysis

Experimental values were expressed as means ± SEM. Statistical comparisons between two groups would be evaluated with Student’s unpaired *t*-test. Statistical analysis of the data among multigroups was performed using the SPSS 19.0 software. Two-way analysis of variance (ANOVA) was applied to analyze difference in data of biochemical parameters among the different groups, followed by Dunnett’s significant post hoc test for pairwise multiple comparisons. Differences were considered as statistically significant at *p* < 0.05.

## Electronic supplementary material

Supplementary information
