# Author Correction: Ethyl Acetate Extract Components of Bushen-Yizhi Formula Provides Neuroprotection against Scopolamine-induced Cognitive Impairment

**DOI:** 10.1038/s41598-020-57439-3

**Published:** 2020-01-15

**Authors:** Shi-Jie Zhang, Dan Luo, Lin Li, Rui-Rong Tan, Qing-Qing Xu, Jie Qin, Lei Zhu, Na-Chuan Luo, Ting-Ting Xu, Rong Zhang, Lei Yang, Qi Wang

**Affiliations:** 1 Institute of Clinical Pharmacology, Guangzhou University of Chinese Medicine, Guangzhou, China; 2 International Center for Translational Chinese Medicine, Sichuan Academy of Chinese Medicine Sciences, Chengdu, China; 3 Department of Radiology, the Third Affiliated Hospital, Sun Yat-sen University, Guangzhou, China

Correction to: *Scientific Reports* 10.1038/s41598-017-10437-4, published online 29 August 2017

In Figure 6A, the DON group was incorrect. The correct Figure 6 appears below as Figure 1.

**Figure 1:**
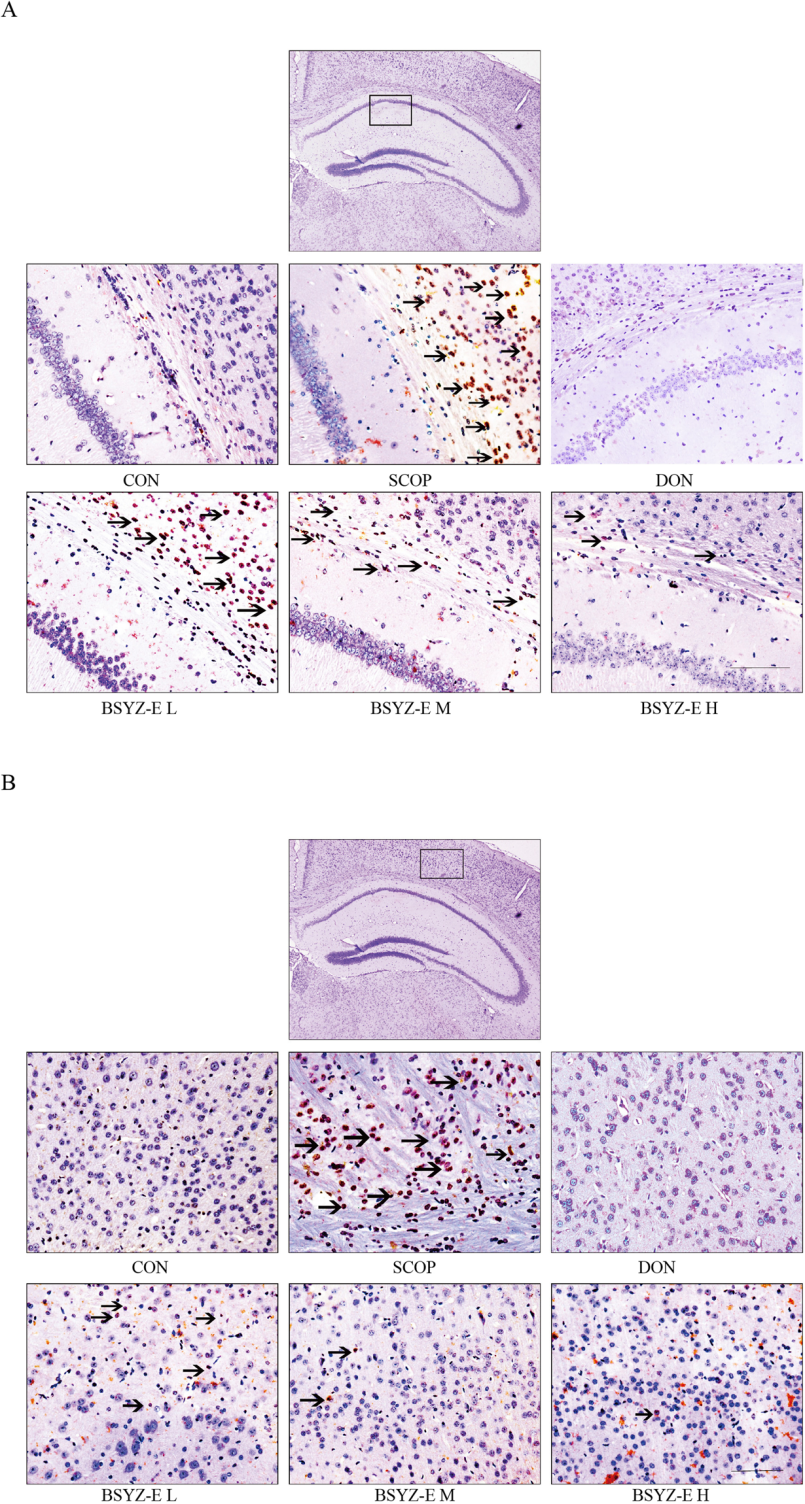
.

