# Supplementary material for: Ethyl Acetate Extract Components of Bushen-Yizhi Formula Provides Neuroprotection against Scopolamine-induced Cognitive Impairment
Source: Sci Rep. 2017 Aug 29;7:9824. doi: 10.1038/s41598-017-10437-4 (PMC5575143; doi:10.1038/s41598-017-10437-4)
Supplement: Supplementary file 1 — Supplementary information [file 41598_2017_10437_MOESM1_ESM.pdf]

Ethyl Acetate Extract Components of Bushen-Yizhi Formula Provides Neuroprotection against Scopolamine-induced Cognitive Impairment

Shi-Jie Zhang[1,*], Dan Luo[1,*], Lin Li[1,*], Rui-Rong Tan[2], Qing-Qing Xu [1], Jie Qin[3], Lei Zhu[1], Na-Chuan Luo[1], Ting-Ting Xu[1], Rong Zhang[1], Lei Yang[1] & Qi Wang[1]

[1]Institute of Clinical Pharmacology, Guangzhou University of Chinese Medicine, Guangzhou, China

[2]International Center for Translational Chinese Medicine, Sichuan Academy of Chinese Medicine Sciences, Chengdu, China

[3]Department of Radiology, the Third Affiliated Hospital, Sun Yat-sen University, Guangzhou, China.

*These authors contributed equally to this work.

Correspondence and requests for materials should be addressed to L.Y. (email: yanglei@gzucm.edu.cn) or Q.W. (email: wangqi@gzucm.edu.cn)

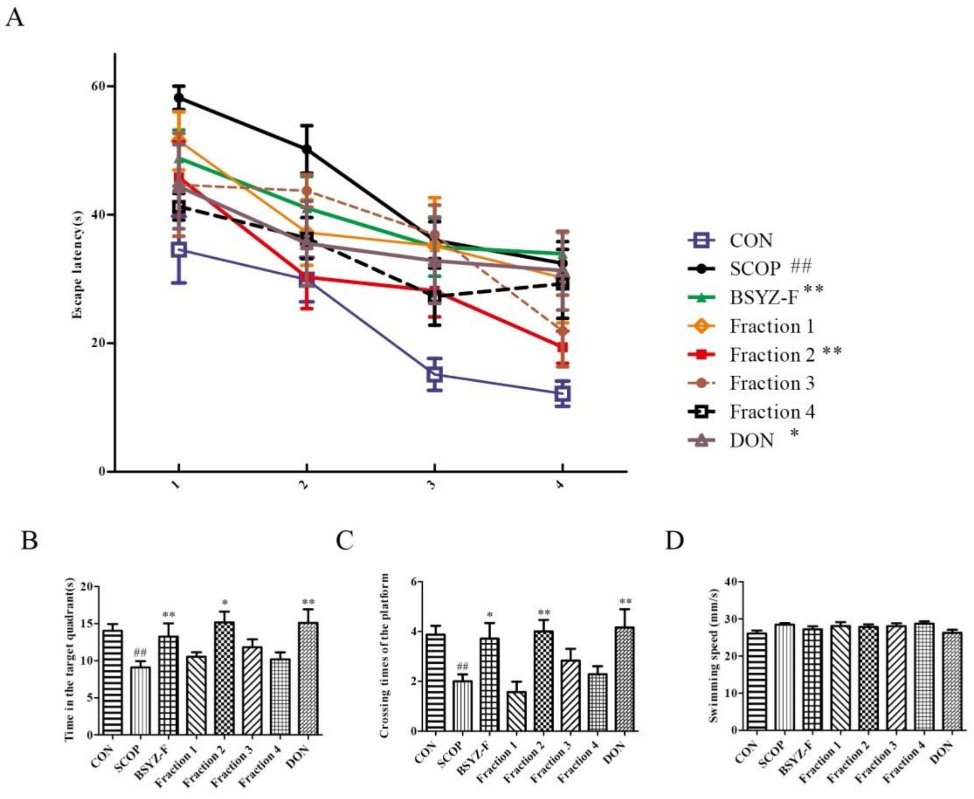

Figure S1. Effects of different extracts of BSYZ-F on cognitive impairment in SCOP-induced mice. Mice were randomly divided into eight groups: CON group, SCOP group, BSYZ-F group, petroleum ether extracts of BSYZ-F group (Fraction 1 group), ethyl acetate extracts of BSYZ-F group (Fraction 2 group), n-butanol extracts of BSYZ-F group (Fraction 3 group), ethanol extracts of BSYZ-F group (Fraction 4 group), and Don group. The Morris water maze (MWM) test was performed following a defined method (**A-D**). Escape latency of four consecutive days' test (**A**), time spent in the target quadrant (**C**), the crossing times of the platform (**D**) and average swimming speed (**E**) are shown. BSYZ-F, Fraction2 and DON treatment mice have shown significant neuroprotection against SCOP-induced cognition dysfunction. *$p < 0.05$, $^{\#\#}p < 0.01$ versus CON group. *$p < 0.05$, *$p < 0.01$ versus SCOP group.

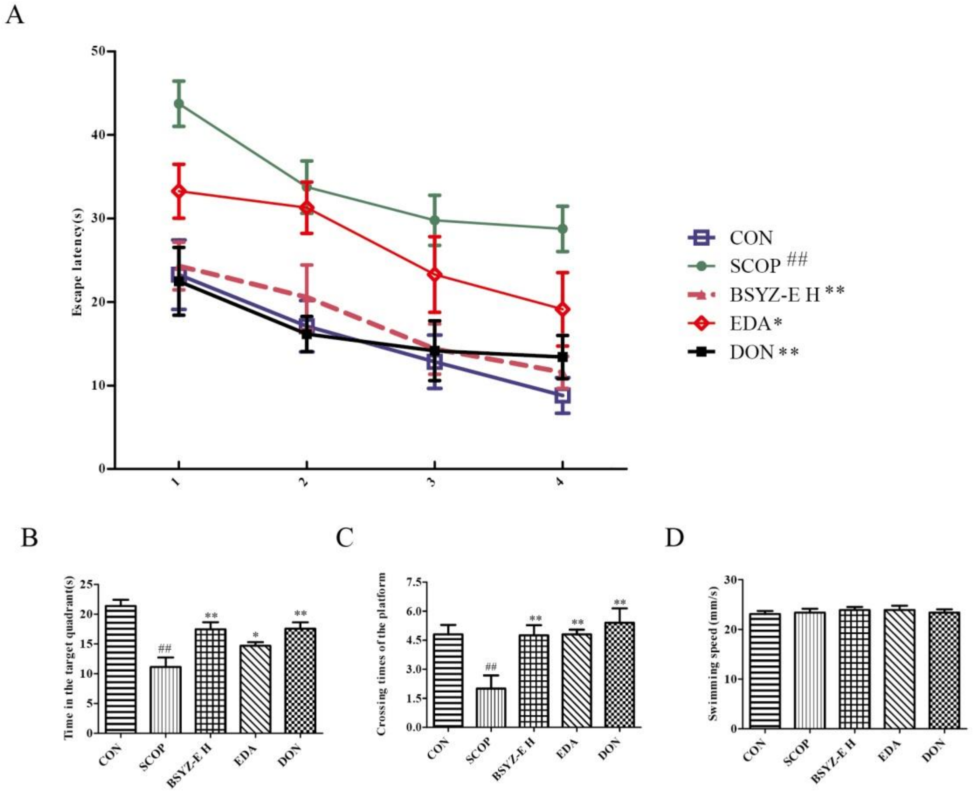

Figure S2. BSYZ-E and Edaravone provides neuroprotection effects on cognitive impairment in SCOP-induced mice. Mice were randomly divided into five groups: CON group, SCOP group, BSYZ-E H group, Edaravone group (EDA group) and Don group. The Morris water maze (MWM) test was performed According to the previous test method (**A-D**). Escape latency of four consecutive days' test (**A**), time spent in the target quadrant (**C**), the crossing times of the platform (**D**) and average swimming speed (**E)** are shown. BSYZ-F, EDA and DON treatment mice have shown significantly neuroprotection against SCOP-induced cognition dysfunction. *p < 0.05, $^{\#\#}p$<0.01 versus CON group. *p <0.05, *p<0.01 versus SCOP group.
